# Supplementary material for: Total, Added, and Free Sugar Consumption and Adherence to Guidelines in Switzerland: Results from the First National Nutrition Survey menuCH
Source: Nutrients. 2019 May 19;11(5):1117. doi: 10.3390/nu11051117 (PMC6566881; doi:10.3390/nu11051117)
Supplement: Supplementary file 1 [file nutrients-11-01117-s001.pdf]

Supplementary Material

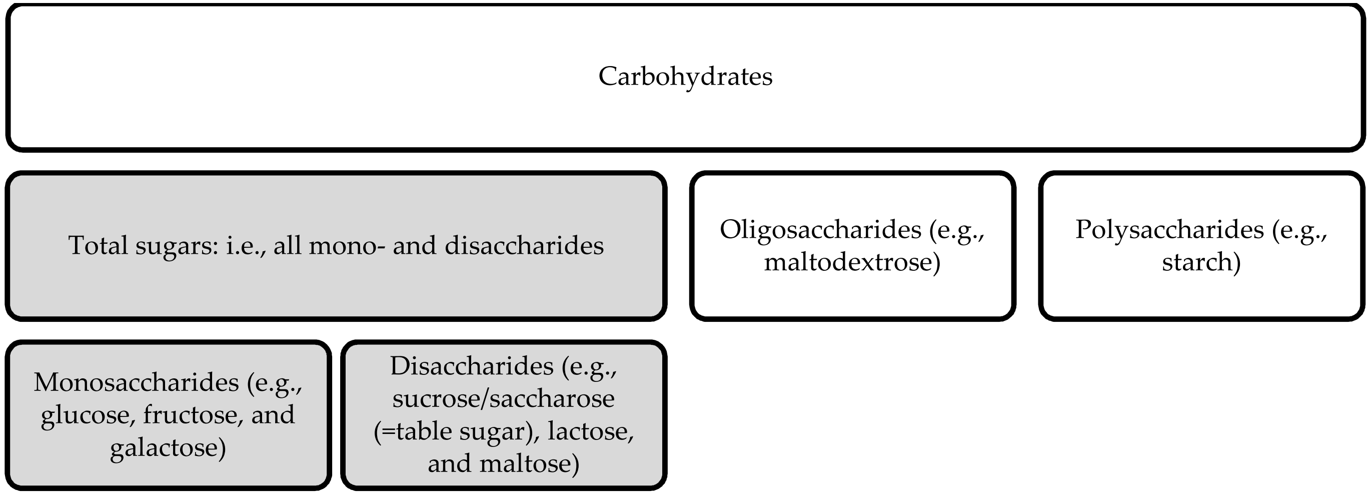

Figure S1. Overview of the different types of carbohydrates (in this paper, we focus only on monosaccharides and disaccharides, in light grey).

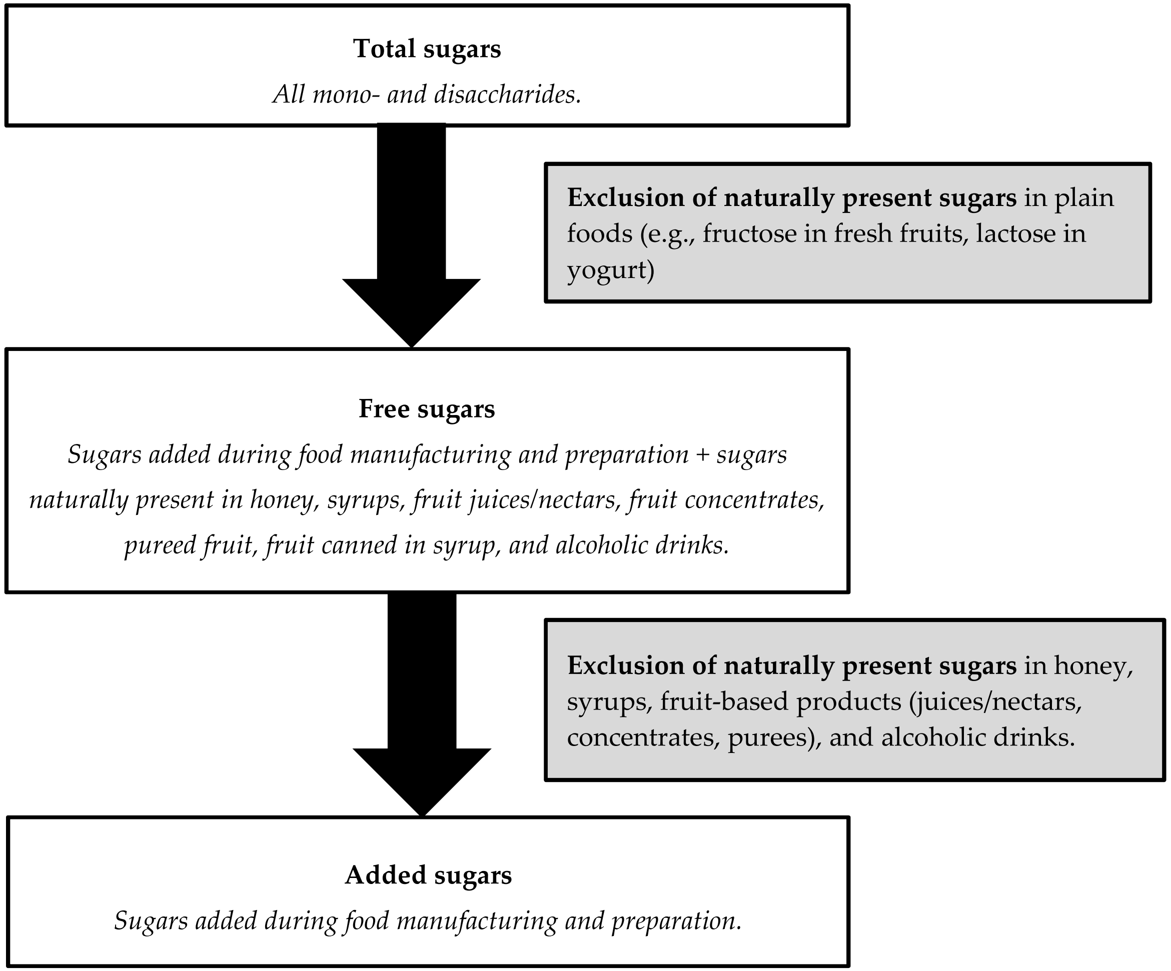

Figure S2. Overview of the estimation of free and added sugars in all consumed foods and beverages.

Table S1. Decision flow chart of estimated percentage for added sugars from total sugars. Free sugars are assumed to be the same as added sugars except where marked with an asterisk (*), in which case 100% of the total sugars were assumed to be free sugars. These decisions were largely based upon the definition by Sluik et al. in the Netherlands, Bowman in the United States (USA), and Swan et al. in the United Kingdom (UK).

Food Group	Decision (% of added sugars from total sugars)	Proportion of all consumed foods[1]
**Tubercles and potatoes products**	Cooked, baked, and mashed potatoes and other tubercles, without sauces or fillings, do not contain added sugars (0%)<br>Processed potatoes (e.g., French fries, rösti, gnocci), without sauces and fillings, may contain a negligible amount of added sugars (50% of total sugars)	1.2
**Vegetables**<br>*Excluding olives, herbs, vegetable juices, or if contained in small amounts in salty snacks, bread, or sauces (e.g., ketchup, pesto sauce)*	Fresh, boiled, baked, and dried vegetables and mushrooms, without sauces or fillings, do not contain added sugars (0%)<br>Canned vegetables and mushrooms may contain a negligible amount of added sugars (20% of total sugars)<br>Pickled and marinated vegetables and mushrooms may contain a negligible amount of added sugars (50% of total sugars)	11.2
**Fruit**<br>*Excluding fruit juices, fruit jams, candied fruits or if contained in small amounts in yogurts, ice cream, and other sweets*	Fresh, boiled, baked, and dried fruits, fruits canned in their own juice, and fruit and vegetable salads do not contain added sugars (0%)<br>Fruits canned in syrup contain 30% added sugars (estimation based on total sugars minus natural occurring sugars in fruits canned without syrup, with pineapple slices as a reference)*<br>Fruit-based puree and compote contain 15% added sugars (estimation based on total sugars minus natural occurring sugars in an unsweetened fruit-based puree and compote, with apple puree as a reference)*	5.1
**Nuts, seeds, and olives**<br>*Excluding peanut butter, nuts coated with chocolate or caramel*	Natural, roasted, and/or salted nuts and seeds do not contain added sugars (0%)<br>Olives are often conserved in a liquid that may contain a negligible amount of added sugars (20% of total sugars)	1.3
**Milk**	Plain milk does not contain added sugars (0%)<br>Sweetened condensed milk contains 80% added sugars (estimation based on total sugars minus natural occurring sugars in unsweetened condensed milk as a reference)	3.8
**Dairy beverages**	Flavored, fruited, and sweetened dairy beverages (e.g., probiotic shots, shakes with fermented milk, milkshakes) contain 20–70% added sugars (estimation based on total sugars minus natural occurring sugars in plain dairy beverages)	0.2
**Dairy desserts**	Flavored, fruited, and sweetened dairy desserts (e.g., custard, chocolate mousse, panna cotta) contain 60–85% added sugars (estimation based on total sugars minus natural occurring sugars from milk (powder))	0.2

**Yogurt**	Plain and light yogurts do not contain added sugars (0%)<br>Flavored and sweetened yogurts (e.g., vanilla, chocolate, coffee) contain 50–70% added sugars (estimation based on total sugars minus natural occurring sugars in plain yogurt)<br>Fruited and sweetened yogurts (e.g., strawberry, raspberry) contain 45–65% added sugars (estimation based on total sugars minus natural occurring sugars in plain yogurt)	1.3
**Cottage cheese, quark**	Plain and light quarks, cottage cheese, and ricotta do not contain added sugars (0%)<br>Flavored, fruited, and sweetened quarks (e.g., chocolate, strawberry, banana) contain 70–75% added sugars (estimation based on total sugars minus natural occurring sugars in plain quark)	0.3
**Bread, bread products, and dough**<br>*<u>Excluding</u> stuffed croissants (e.g., hazelnut croissants) and sweet pastries (e.g., chocolate croissants)*	Plain breads, bread products, and dough with a low total sugar value (≤3 g, e.g., most white and wholegrain bread, flat bread, toast bread, dough for pizza) and with unsweetened fruit (e.g., bread with sultanas) contain a negligible amount added sugar (0%)<br>Added sugars in bread products with a small amount of milk and nuts or with a high total sugar value (>3 g, e.g., dried bread, croissants, sweet bread, and rolls (e.g., brioche), sweetened dough for pie) are assumed to be equal to total sugars minus 3 g of natural sugars from plain bread, milk, and/or nuts	6.1
**Breakfast cereals**	Branded breakfast cereals without dried fruit and nuts contain 99% added sugars (estimation based on the assumption that there is 1% of natural sugar in grains and cereals), except when specified as without added sugar (0%)<br>Branded breakfast cereals and branded muesli mixes with dried fruit and nuts contain 75–90% added sugars (estimation based on total sugars minus natural occurring sugars in dried fruits and nuts and on the assumption that there is 1% of natural sugar in grains and cereals), except when specified as without added sugar (0%)	0.6
**Other starchy foods, cereals, and legumes**	Plain flours do not contain added sugars (0%)<br>Plain flakes (e.g., oat, wheat) do not contain added sugars (0%)<br>Plain grains (e.g., rice, corn, quinoa) do not contain added sugars (0%)<br>Plain pasta (e.g., spaghetti, tagliatelle) do not contain added sugars (0%)<br>Stuffed pasta (e.g., tortellini, ravioli) may contain a negligible amount of added sugars (20% of total sugars)<br>Plain legumes do not contain added sugars (0%)<br>Canned legumes (e.g., beans in tomato sauce) may contain a negligible amount of added sugars (20% of total sugars)	3.2
**Sugar**	Table sugar (e.g. white, brown) contain 100% added sugars	2.3
**Honey, jam, and other sweet sauces/spreads**	Honey contain 100% added sugars<br>Fruit jams and jelly contain 85% added sugars, except lemon curd (95%) and milk-based jam (90%) (estimation based on total sugars minus natural occurring sugars in fruit and/or milk)*<br>Sweet spreads (e.g., chocolate spread) and sweet toppings (e.g., caramel or chocolate sauce) contain 95–100% added sugars, except peanut butter (70%)<br>Chocolate/cacao powder contain 95% added sugars, except when specified as without added sugar (0%)	2.1

**Confectionary and candies**	All sweets and candies (e.g., lollipop, marshmallows, candied fruit, toffee, nuts coated with chocolate or caramel, and nougat) contain 90–100% added sugars<br>Chocolate products and confectionary made with chocolate (e.g., filled chocolate, pralines, branded chocolate bars) contain 85–99% added sugars (estimation based on total sugars minus natural occurring sugars in milk powder and/or nuts)<br>Sweet and sports cereal bars (e.g., energy bars) contain 90–95% added sugars (estimation based on total sugars minus natural occurring sugars in dried fruit), except when specified as without added sugar (0%)	1.1
**Pure chocolate**	White and milk chocolate contain 95% added sugars (estimation based on total sugars minus natural occurring sugars in milk (powder))<br>Dark chocolate contain 99% added sugars (estimation based on total sugars minus natural occurring sugars in plain cocoa)	0.7
**Syrups**	Syrups (e.g., maple syrup) contain 100% added sugars<br>Fruit-based syrup concentrates (to be diluted in water) contain 98% added sugars*	0.2
**Ice-cream**	Flavored, fruited, and sweetened milk-based or cream-based ice creams contain 75–95% added sugars (estimation based on total sugars minus natural occurring sugars in fruit and/or milk (powder))<br>Water-based ice creams and sorbets contain 90–100% added sugars (estimation based on total sugars minus natural occurring sugars in fruit)*	0.4
**Cakes and cookies**<br>*Excluding fruit-based pies: individual ingredients (e.g., fruit, white sugar, dough) were split into different food groups*	Sweet cakes and pastries (e.g., brownies, lemon cakes, muffins, and Linzer tart), and sweet pastries (e.g., chocolate croissants, doughnuts, and waffles) contain 90–100% added sugars (estimation based on total sugars minus natural occurring sugars in milk (powder) and/or nuts). Examples: brownies (95%), chocolate and lemon cakes (99%), muffins (98%), chocolate croissant (93%)<br>Fruit-filled pastries (e.g., French apple turnovers) contain 30–80% added sugars (estimation based on total sugars minus natural occurring sugars in fruit, milk (powder) and/or nuts)<br>Sweet and dried biscuits (e.g., shortbread, amaretto, chocolate chip cookies) contain 80–100% added sugars (estimation based on total sugars minus natural occurring sugars in fruit, milk (powder) and/or nuts). Examples: meringue (100%), shortbread (90%), and chocolate biscuit (88%)	2.0
**Fruit and vegetable juices***	Juices made of 100% fruit and/or vegetable and smoothies do not contain added sugars (0%)*<br>Fruit concentrates do not contain added sugars (0%), because it is assumed that fruit concentrates will be diluted to the original ratio of fruit juices before consumption*<br>'Schorle' (i.e. fruit juices, often apple juice, mixed with water) does not contain added sugars (0%)*<br>Fruit drinks and nectars contain 30–50% added sugar*	1.8
**Soft drinks**	100% sugar-free and light soft drinks do not contain added sugars (0%)<br>Sweetened soft drinks, sports and energy drinks, fizzy drinks, ice tea, industrialized lemonades, and diluted syrup contain 85–100% added sugar<br>Alcoholic drink substitutes (e.g., champagne substitute for children, hot punch for Christmas) contain 100% added sugar, except alcohol-free beers (0%)	1.7
**Water**	Water does not contain added sugars (0%), except flavored and sweetened water (100%)	10.8

**Coffee and tea**	Tea and coffee do not contain added sugars (0%), except when indicated (100%)<br>Coffee mixes (e.g., cappuccino, macchiato) contain 0–80% added sugars (estimation based on total sugars minus natural occurring sugars in plain milk)	9.1
**Alcoholic drinks***	Beers do not contain added sugars (0%)*<br>Ciders do not contain added sugars (0%)*<br>White and red wines, champagne, port wines, sherry, vermouths do not contain added sugars (0%)*<br>Dry spirits and liquors (e.g., vodka, rum, and whiskey) do not contain added sugars (0%)*<br>Wine products (e.g., sangria, hot punch for Christmas with alcohol) contains 95–100% added sugars (estimation based on standard recipe)*<br>Shandy, cocktails and long drinks, liqueurs, punch, 'alcopops', and any other alcoholic drinks (e.g., coffee with spirit) contains 90–100% added sugars (estimation based on standard recipe)*	2.4
**Processed meat**	Transformed and canned meat (e.g., chicken nuggets, sausages, cold cuts, beef jerky, and pâté) contain 100% added sugars	2.6
**Processed fish and seafood**	Fish and seafood products (e.g., fish in crumbs, fish mousse, and surimi) contain 100% added sugars	0.1
**Sauces, condiments, and flavorings**	White sauces (e.g., mayonnaise), cream-based sauces (e.g., Béchamel), brown sauces for meat (e.g., gravy), and other sauces (e.g., curry sauce, soya sauce, BBQ sauce, and sweet-sour sauces) contain 50–100% added sugars<br>Salad dressings contain 50–70% added sugars<br>Fruit-based and nut-based sauces (e.g., chutney, satay sauce) contain 90% added sugars*<br>Water-based and oil-based vegetable sauce (e.g., tomato sauce, pesto, and tapenade) may contain a negligible amount of added sugars (20% of total sugars)<br>Ketchup contains 85% added sugars<br>Unsweetened sambal/chilli sauce (e.g., Tabasco) does not contain added sugars (0%)<br>Mustard contains 65% added sugars<br>Yeast does not contain added sugars (0%)<br>Salt does not contain added sugars (0%)<br>Vinegar does not contain added sugars (0%), except balsamic vinegar (80%)<br>Plain spices (e.g., cinnamon, pepper) and herbs (e.g., basil, fresh mint) do not contain added sugars (0%)	10.2
**Soups**	Handmade soups do not contain added sugars (0%)<br>Non-handmade or instant soups may contain a negligible amount of added sugars (50% of total sugars)<br>Stock contains 100% added sugars	0.7

**Salty snacks**	Dried salty crackers (e.g., crisps, sticks, pretzels, and shrimp crackers) and prepared salty snacks (e.g., cocktail canapés, spring rolls, ham croissants, and sausage rolls) may contain a negligible amount of added sugars (50% of total sugars)	0.6
**Others/miscellaneous**	All types of cheese (e.g., soft, hard) and branded spread cheese do not contain added sugars (0%)	16.9
	Unprocessed meat, poultry, game/venison, and offals do not contain added sugars (0%)	
	Fresh and cooked fish, seafood, and amphibians (e.g., frog) do not contain added sugars (0%)	
	Canned fish (e.g., canned haring) may contain a negligible amount of added sugars (50% of total sugars)	
	Eggs consumed as such do not contain added sugars (0%)	
	Stevia and artificial sweeteners do not contain added sugars (0%)	
	Plain fats and oils (e.g., butter, olive oil) do not contain added sugars (0%)	
	Plain cream do not contain added sugars (0%), except sweetened whipped cream (50–70% added sugars, estimation based on total sugars minus natural occurring sugars in plain cream)	
	Vegetable substitutes of yogurt (e.g., soya yogurts) contain 85% added sugars.	
	Vegetable substitutes of milk and cream (e.g., rice milk, coconut milk) may contain a negligible amount of added sugars (20% of total sugars)*	
	Vegetable substitutes of meat (e.g., tofu, quorn, vegetarian nuggets, falafel) may contain a negligible amount of added sugars (20% of total sugars)	
	Dietetic products (e.g., protein bars and shakes) may contain added sugars, but are a negligible source of added sugars in the general population (50% of total sugars)	
	Others (e.g., gelatin, pollen) do not contain added sugars (0%)	

[1] The proportions are based on the number of consumed/cited foods for a defined food group divided by the total number of consumed/cited foods: e.g., out of the 121,047 consumed/cited foods in total in the survey, 1432 were classified in the food group ‘Tubercles and potatoes products’, representing 1.2% (1432/121,047 = 1.2%). * These products were assumed to contain 100% free sugars; for all other food items, added sugars are assumed to be equal to free sugars. Mixed dishes (e.g., sandwiches, pizza, composed salads, sushi, salty crepes/omelettes and sweet crepes/pancakes, paella, hamburger, and kebab sandwiches) are disaggregated into ingredients.
